# Ovulation Prevalence in Women with Spontaneous Normal-Length Menstrual Cycles – A Population-Based Cohort from HUNT3, Norway

**DOI:** 10.1371/journal.pone.0134473

**Published:** 2015-08-20

**Authors:** Jerilynn C. Prior, Marit Naess, Arnulf Langhammer, Siri Forsmo

**Affiliations:** 1 Centre for Menstrual Cycle and Ovulation Research, University of British Columbia, Vancouver, Canada; 2 Division of Endocrinology, Department of Medicine, University of British Columbia, Vancouver, Canada; 3 School of Population and Public Health, University of British Columbia, Vancouver, Canada; 4 Vancouver Coastal Health Research Institute, Vancouver, Canada; 5 HUNT Research Centre, Department of Public Health and General Practice, Norwegian University of Science and Technology, Levanger, Norway; 6 Department of Public Health and General Practice, Norwegian University of Science and Technology, Trondheim, Norway; Central South University, CHINA

## Abstract

**Background:**

Ovulatory menstrual cycles are essential for women’s fertility and needed to prevent bone loss. There is a medical/cultural expectation that clinically normal menstrual cycles are *inevitably ovulatory*. Currently within the general population it is unknown the proportion of regular, normal-length menstrual cycles that are ovulatory. Thus, the objective of this study was to determine the population point prevalence of ovulation in premenopausal, normally menstruating women. The null hypothesis was that such cycles are ovulatory.

**Methods:**

This is a single-cycle, cross-sectional, population-based study—a sub-study of the HUNT3 health study in the semi-rural county (Nord Trøndelag) in mid-Norway. Participants included >3,700 spontaneously (no hormonal contraception) menstruating women, primarily Caucasian, ages 20–49.9 from that county. Participation rate was 51.9%. All reported the date previous flow started. A single, random serum progesterone level was considered ovulatory if ≥9.54 nmol/L on cycle days 14 to -3 days before usual cycle length (CL).

**Results:**

Ovulation was assessed in 3,168 women mean age 41.7 (interquartile range, [IQR] 36.8 to 45.5), cycle length 28 days (d) (IQR 28 to 28) and body mass index (BMI) 26.3 kg/m2 (95% CI 26.1 to 26.4). Parity was 95.6%, 30% smoked, 61.3% exercised regularly and 18% were obese. 1,545 women with a serum progesterone level on cycle days 14 to -3 were presumed to be in the luteal phase. Of these, 63.3% of women had an ovulatory cycle (n = 978) and 37% (n = 567) were anovulatory. Women with/ without ovulation did not differ in age, BMI, cycle day, menarche age, cigarette use, physical activity, % obesity or self-reported health. There were minimal differences in parity (96.7% vs. 94.5%, P = 0.04) and major differences in progesterone level (24.5 vs. 3.8 nmol/L, P = 0.001).

**Conclusion:**

Anovulation in a random population occurs in over a third of clinically normal menstrual cycles.

## Introduction

Regular, normal length (21–35 days [1]) menstrual cycles are considered a vital sign representing women’s wellness [2]. Some consider regular menstruation sufficient evidence for ovulation [3] and thus the production of normal progesterone as well as estradiol levels. Ovulation is important because both ovulation and sufficient luteal phase lengths (duration of progesterone production) are necessary for fertility. Younger women are more commonly anovulatory [4], as are those in the menopause transition [4–6]. Studies in healthy, highly screened premenopausal women suggest that 92–97% of regular cycles are ovulatory [7–9]. However, regular menstrual cycles with normal estradiol levels may lack ovulation [10], due to hypothalamic adaptations related to nutritional, energetic, socioeconomic and emotional stressors that women in the population commonly experience [11].

The gold standard for ovulation documentation is direct visualization of an egg being extruded from the ovary, but many *indirect* methods show validated evidence of ovulation, including urinary progesterone excretion (pregnanediol, PdG)[12,13], the midcycle luteinizing hormone (LH) peak [14] and salivary or serum progesterone [15] levels. The quantitative effect of progesterone to raise core temperature is also utilized to document ovulation and luteal phase lengths [16,17]. One group, however, considered high post-ovulatory progesterone levels to be “an endocrine/metabolic disorder unique to young women”[18]. Evidence suggests that silent anovulation within normal-length cycles during the premenopausal years is associated with common diseases of older women including osteoporosis [19], cardiovascular disease [20] as well as breast [21] and endometrial [22] cancers.

Population-based large studies of ovulation prevalence are needed to determine whether ovulation is invariably present or, alternatively, is a common subclinical problem within regular cycles. The few available random population epidemiological studies in less than 1000 women in total show percentage ovulation prevalences ranging from 73 [20] to 74.3 [23] to 84.4% [24]. An ongoing whole-county health study in Norway (HUNT) afforded an opportunity to ascertain population ovulation prevalence. Thus, the purpose of this study was to cross-sectionally document ovulation prevalence in a population of spontaneously menstruating premenopausal women by measuring a single cycle-day documented progesterone level. Our null hypothesis was that regular, normal-length menstrual cycles are ovulatory.

## Methods

### Study design and enrolment

HUNT3 is the 2006–8 re-examination of the population [25] in a multipurpose health study in Nord Trøndelag, a semi-rural county with a population of about 132,000. It assessed adults ≥20 years of age (overall participation rate 54.1%) [25]. A mailed invitation included comprehensive general health and lifestyle questionnaires and the date for an examination. At the visit, women provided the date of their last menstrual period (LMP), blood samples and completed additional questionnaires; standardized measurements of blood pressure, height (in centimeters, cm) without shoes and weight (in kilograms, kg) in light clothing were collected. The menstrual cycle day of the blood sample was defined by LMP.

### Participants

A total of 47,293 women were invited and 27,754 women (58.8%) attended. The study cohort totaled 3709 spontaneously menstruating women ages 20–49.9 after excluding women ≥50 years and those using hormonal contraception or the Levonorgestrel-impregnated intrauterine device (LNG-IUD). An approximate 10% sample (n = 949) was also provided a form to return the start date of their next menstrual period (NMP) in a postage-paid envelope (**S1 Protocol**).

Women were excluded if currently using hormonal contraception including a progestin-releasing IUD, if they were menopausal, perimenopausal with irregular or abnormal-length cycles, had a hysterectomy, were immediately post-partum or had lactational amenorrhea. All women were additionally asked: “Have you had regular periods during the last 12 months?” Women were excluded who answered “no” (irregular cycles) or who were regularly cycling but with a reported usual CL <21 d (n = 15) or >35d (n = 10) [1] or had missing CL data (n = 62).

All women signed informed consent; this study was approved by the Regional Committee for Medical Research Ethics, the Norwegian Data Inspectorate and the Clinical Research Ethics Board of the University of British Columbia (because of funding by the Canadian Institutes for Health Research, #H06-00204).

### Outcome measures

In an open-ended question, all women were asked to record their usual cycle length (CL) within the last 12 months as a two-digit, specific number of days. They were also asked to record the date their last flow started (LMP). The primary outcome was the cycle-timed serum progesterone threshold level for evidence of ovulation of ≥9.54 nmol/L [15,26]. Because of uncertainty within the literature and in the medical community, we choose published experts from three countries and asked them this open-ended question: “If you had a cycle-day related single serum progesterone level in a population-based cohort of premenopausal regularly menstruating women, *What progesterone threshold would you suggest using to make a diagnosis of an ovulatory cycle*?”The majority (four of five) of reproductive clinical and scientific experts spontaneously recommended a progesterone threshold of ≥9.54 nmol/L (≥3.0 ng/mL); a single expert recommended 19.1 nmol/L (6.0 ng/mL). Other potential progesterone ovulation thresholds are also possible; one excludes the follicular phase (≥8.0 nmol/L)[27] and others are as low as ≥3.5 nmol/L. Those cycles with indirect hormonal evidence of ovulation are called ovulatory and those without this are called anovulatory. Based on the date women’s last flow began (last menstrual period, LMP), a date that women had been instructed to record/remember in their invitation letter, cycle days 14 to 3 days before the woman’s usual cycle length (CL) were presumed to be the luteal phase. The progesterone threshold of ≥9.54 nmol/L was used to provide evidence of ovulation in sera collected during these cycle days. These were also the presumed luteal phase based on the cohort’s cycle-day distribution of progesterone levels and on assessment of the population’s cycle-day likelihood of exceeding the progesterone threshold [27].

Progesterone was analyzed by a direct competitive chemiluminescence immunoassay (CV 4.6% at 74.7 nmol/L) with a listed luteal phase range of 3.8 to 78.9 nmol/L (DiaSorin, Sundbyberg, Sweden). A state-of-the-art Biobank processed and stored blood fractions [25,27]. Serum collection, storage and progesterone and estradiol analyses are reported in **S2 Protocol**.

Reproductive variables were collected by self- and interviewer-administered questionnaires. Women reported their age at menarche, parity (borne a child) and numbers of live births. They additionally reported history of hormonal contraceptive and LNG-IUD use, amenorrhea (≥3 months without flow), infertility (>12 months without conception), lactation, if they were menopausal and if they had experienced hysterectomy and/or removal of one/both ovaries.

The general health questionnaire included body mass index (BMI) at age 18; history of cigarette use as current, past or never; alcohol servings per 2-weeks and the frequency of physical activity, its duration and intensity. Current self-rated health was reported in four categories (poor, fair, good and very good /excellent health). For purposes of analysis, pre-obese (BMI = 25–29.9 kg/m^2^) and obesity (BMI≥30 kg/m^2^) were defined according to WHO criteria. The history of prior use of hormonal contraception was dichotomized into never and ever users; exercise frequency was dichotomized into <2 and ≥2 hours per week; current self-rated health was re-coded into two categories: fair and good. Further categorizations for the multivariable analyses were age (groups of 5 years above the age of 30), grouped cycle days (≤16, 17–20, 21–24, ≥25) and serum estradiol (≤120.0, 121.0–299.0, 300.0–499.0 and >500.0 pmol/L) levels.

### Statistical Analyses

A progesterone threshold of ≥9.54 nmol/L, the primary outcome, was the dependent variable in bi- and multi-variable analyses; other progesterone thresholds (≥3.5, ≥8.0 and ≥19.1 nmol/L) were secondarily also assessed. Sensitivity analysis examined the cohort reporting both LMP and NMP dates. The analyses were performed by appropriate data distribution-related parametric or non-parametric methods. Baseline differences were tested by independent sample t-test, Mann-Whitney U test or Chi-square tests. The odds ratio for ovulation was calculated by logistic regression in univariable and multivariable models among women in the presumed luteal phase; significant univariate predictors were included in the multivariable logistic regression models. The final model was also assessed for interaction terms. All statistical tests were two-sided; analyses were performed with SPSS (IBM, Armonk, NY, USA) version 20.

## Results

**Fig 1:**
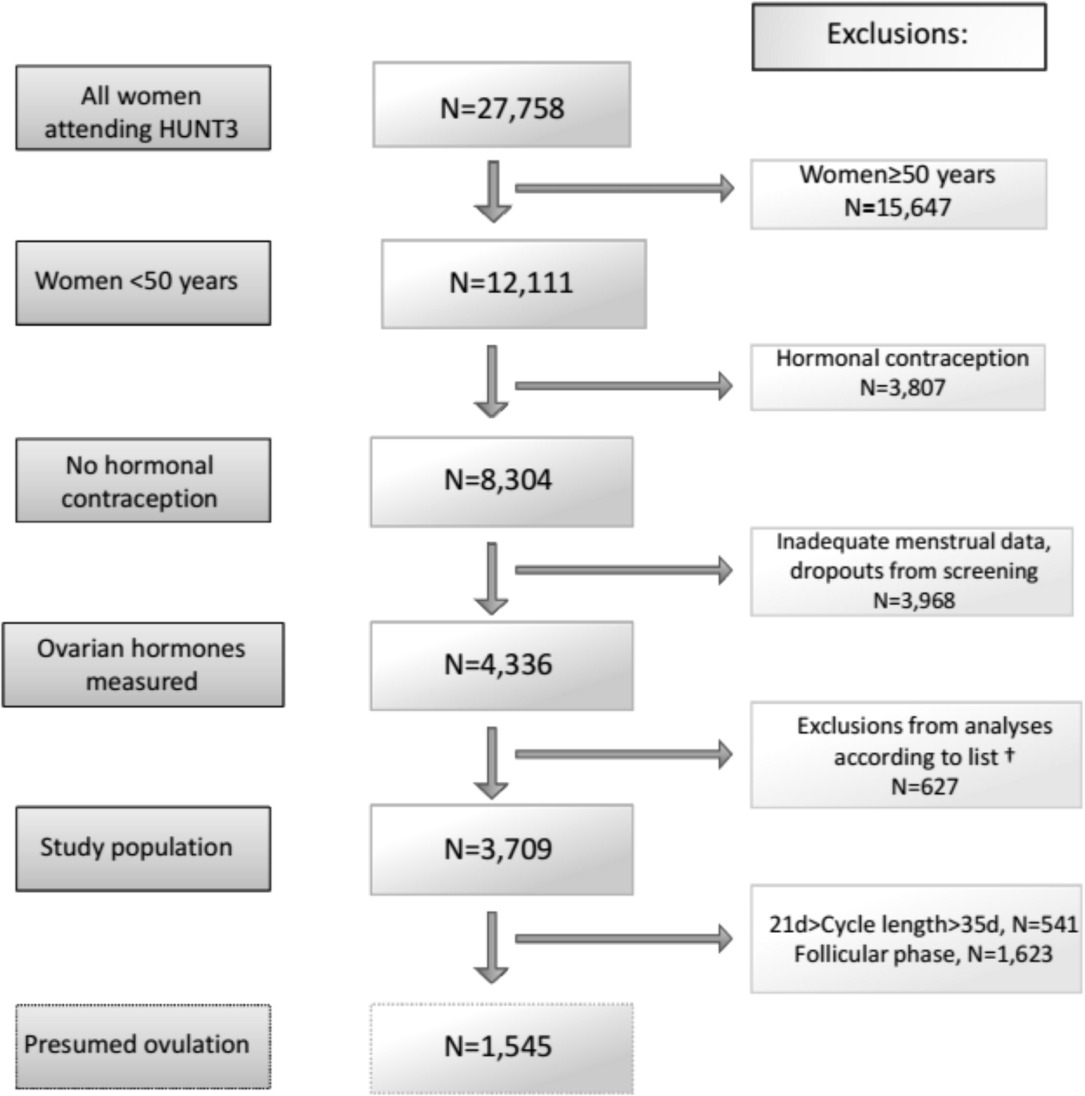
Consort-like flowchart of women in the third Nord Trøndelag Health Study (HUNT3, Norway) population-based cohort for assessment of the ovulation point prevalence. † Indicates women excluded due to pregnancy, childbirth within the last year, hysterectomy with or without single or bilateral ovariectomy, probable menopause, or missing data.

Participant flow through this population-based examination of ovulation point prevalence is shown in **Fig 1**. The age-cohort participation rate was 51.9% among the 12,111 women younger than 50. After exclusion of hormonal contraception, those dropping out and those with incomplete data, 4,336 women with a hormonal sample remained. Among the 3,709 spontaneously menstruating women potentially eligible for assessment of ovulation, a total of 3,236 women (87.2%) reported regular cycles and a usual CL. Those included and those with irregular cycles (12.8%) who were excluded are compared in **Table 1.** Excluded women with irregular cycles were significantly older, heavier, more likely to have experienced amenorrhea, to be smokers, to have lower self-reported health and mean cycle levels of progesterone and estradiol.

**Table 1:** Characteristics of 3,709 spontaneously menstruating* women ages 20–49.9 in the third Nord-Trøndelag Health Study (HUNT3), Norway reporting regular or irregular menstrual cycles in the last 12 months.

Characteristics	Regular cycles N = 3,236 (87.2%)		Irregular cycles N = 473 (12.8%)		
	Mean/Median/%	95% CI /(IQR)	Mean/Median/%	95% CI /(IQR)	P value
**Age (median)**	41.6	(36.8 to 45.5)	41.5	(34.9 to 47.1)	0.7
**≥45 years (%)**	29.0%	27.4 to 30.6	36.2%	32.0 to 40.6	≤0.002
**Weight (kg) (mean)**	72.9	72.4 to 73.4	75.8	74.3 to 77.3	≤0.001
**Height (cm) (mean)**	166.6	166.4 to 166.8	166.7	166.1 to 167.2	0.8
**BMI kg/m** ^**2**^ **(mean)**	26.3	26.1 to 26.4	27.3	26.8 to 27.8	≤0.001
**Obesity (BMI>30 kg/m** ^**2**^ **) %**	18.6%	17.3 to 20.0	26.5%	22.7 to 30.6	≤0.001
**BMI at age 18 kg/m** ^**2**^ **(mean)**	21.3	21.2 to 21.4	21.6	21.3 to 22.0	0.07
**Overweight at age 18 (BMI>25) %**	8.3%	7.3 to 9.4	11.3%	8.4 to 15.1	0.06
**Menarche age (mean)**	13.1	13.0 to 13.1	13.0	12.8 to 13.1	0.1
**Days since first day in last period**	14.1	13.8 to 14.4	-		
**Cycle length, days (mean)**	27.4	27.3 to 27.5	-		
**Parous (≥ 1 child) %**	95.7%	94.9 to 96.3	94.9%	92.6 to 96.6	0.7
**Infertility >12 months %**	16.8%	15.4 to 18.3	22.4%	17.7 to 28.0	0.03
**Amenorrhea >3 months %**	4.9%	4.1 to 5.8	27.6%	22.4 to 33.5	≤0.001
**Ever hormonal contraception %**	88.8%	87.5 to 90.0	90.0%	85.7 to 93.2	0.7
**Good self-reported health %**	81.3%	79.9 to 82.6	65.8%	61.4 to 70.0	≤0.001
**Current smokers %**	29.7%	28.2 to 31.4	45.5%	41.0 to 50.0	≤0.001
**Alcohol (# units in 2 weeks)**	2	(1 to5)	2	(1 to5)	0.998
**Physical activity ≥2 hours/week**	61.3%	59.6 to 62.9	53.6%	49.1 to 58.1	0.03
**Progesterone (nmol/L) (cycle median)**	4.5	(2.7 to17.2)	3.8	(2.7 to7.3)	≤0.001
**Estradiol (pmol/L) (cycle median)**	250.0	(160.0 to 367.1)	216.6	(220.0 to 350.0)	≤0.001

*Spontaneously menstruating means that they were not currently using hormone contraception.

**Fig 2:**
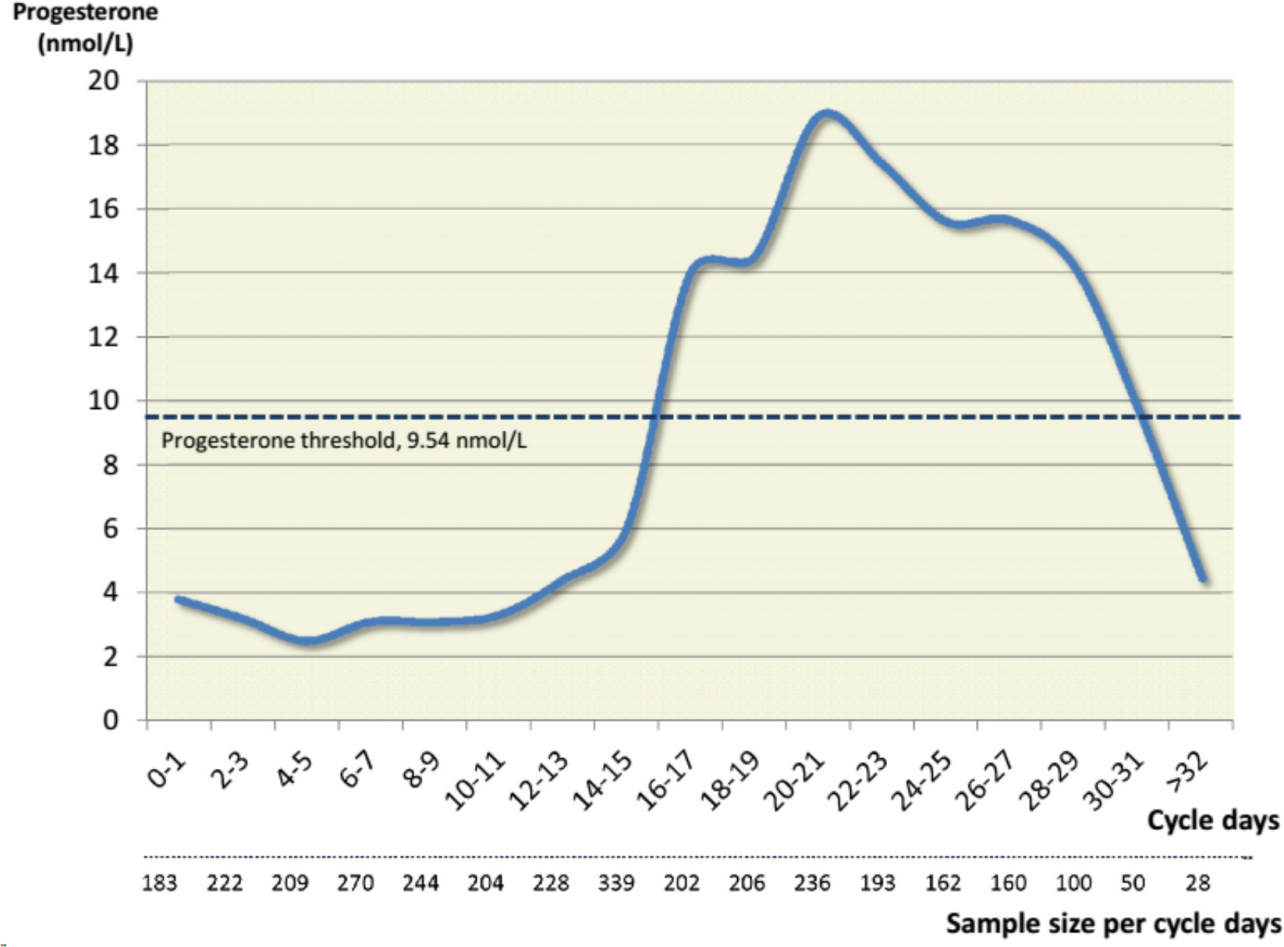
Median serum progesterone levels in nmol/L across a studied menstrual cycle by cycle days in 3236 spontaneously menstruating premenopausal women aged 20–49.9 with regular cycles in HUNT3 (Norway) study.

Median serum progesterone values from all women with regular cycles (n = 3236) are plotted by cycle day in **Fig 2**. The cycle day on which women provided blood samples was randomly distributed; those sampled in cycle days ≤14 or >14 had similar average ages (41.9 versus 41.5), BMI values (26.3 versus 26.2) and mean cycle lengths (27.4 versus 27.4).

The main “ovulation prevalence cohort” of 3,168 women (98% of the regularly cycling cohort) was formed by excluding those not providing a CL or who reported a usual CL outside of the normal range of 21–35 days. A sub-cohort included 307 women who reported start dates for both LMP and NMP; they were clinically identical to the main cohort reporting only LMP. Women in this sub-cohort had slightly higher BMI values (26.8 versus 26.2 kg/m^2^), reported slightly lower menarche ages (12.9 versus 13.1 years) and had somewhat lower self-rated good/excellent health (76.9% versus 81.8%)(data not shown).

**Table 2** documents the characteristics of the women in the presumed luteal phase for whom a serum progesterone was ≥9.54 nmol/L (n = 978, 63.3%) and those with lower levels of progesterone who were considered anovulatory (n = 567, 36.7%) in that cycle. Women in the presumed luteal phase (n = 1545) by cycle day and usual CL had a median progesterone level that was significantly higher (24.5 versus 3.9 nmol/L, p<0.001) than those in follicular/menstrual phases (n = 1623). Apart from differences in median progesterone and estradiol values, the only significant difference was that parity tended to be higher in women with ovulatory cycles (96.7% versus 94.5%, P <0.04); obesity also tended to be less in those who were ovulatory.

**Table 2:** Characteristics of 1545 spontaneously* normally menstruating premenopausal women who, by cycle days and usual cycle length (CL) were in the presumed luteal phase in HUNT3 (Norway) comparing those ovulatory by a serum progesterone threshold of ≥9.54 nmol/L with those without apparent ovulation.

Women in the presumed luteal phase N = 1545	Progesterone ≥9.54 nmol/L N = 978 (63.3%)		Progesterone <9.54nmol/L N = 567 (36.7%)	
	Mean/Median/%	95% CI /(IQR)	Mean/Median/%	95% CI /(IQR)
**Age (median)**	41.8	(37.3 to 45.4)	42.2	(36.6 to 45.8)
**Age>45 (%)**	29.0%	26.3 to 32.0	31.7%	28.1 to 35.7
**Weight (kg) mean**	72.6	71.7 to 73.5	73.3	72.2 to 74.4
**Height (cm) mean**	166.47	166.0 to 166.8	166.3	165.8 to 166.8
**BMI (kg/m** ^**2**^ **)(mean)**	26.2	25.9 to 26.5	26.5	26.1 to 26.9
**Obesity (BMI>30 kg/m** ^**2**^ **) (%)**	16.9%	14.7 to 19.4	20.7%	17.5 to 24.2
**BMI at age 18 (kg/m** ^**2**^ **) mean**	21.3	21.1 to 21.4	21.4	21.2 to 21.7
**Overweight at age 18 (BMI>25) %**	7.2%	5.6 to 9.3	10.0%	7.5 to 13.2
**Menarche age (mean)**	13.1	13.0 to 13.2	13.1	12.9 to 13.2
**Menstrual cycle day (median)**	21	(17 to24)	21	(17 to25)
**Cycle length (mean)**	27.4	27.2 to 27.5	27.4	27.2 to 27.6
**Parous (≥ 1 child) (%)**	96.7%	95.4 to 97.7	94.5%	92.3 to 96.1
**Infertility >12 months (%)**	15.5%	13.0 to 18.2	15.8%	12.6 to 19.6
**Amenorrhea >3 months (%)**	4.5%	3.2 to 6.3	5.8%	3.9 to 8.5
**Ever hormonal contraception %**	90.1%	87.7 to 92.0	90.4%	87.2 to 92.9
**Self-report good health (%)**	82.8%	80.2 to 85.0	81.7%	78.3 to 84.7
**Current smokers %**	29.9%	27.1 to 32.9	29.9%	26.3 to 33.8
**Alcohol (# units in 2 weeks)**	3	(1 to 5)	2	(1 to 5)
**Physical activity≥2 hours/week**	60.5%	57.4 to 63.5	60.7%	56.7 to 64.7
**Progesterone serum (median)nmol/L**	24.5	(17.8 to 32.8)	3.9	(2.4 to 5.7)
**Estradiol serum (median) pmol/L**	290.0	(220 to 370)	230.0	(130 to 420.0)

*Spontaneously menstruating means that they were not currently using hormonal contraception.

**Fig 3:**
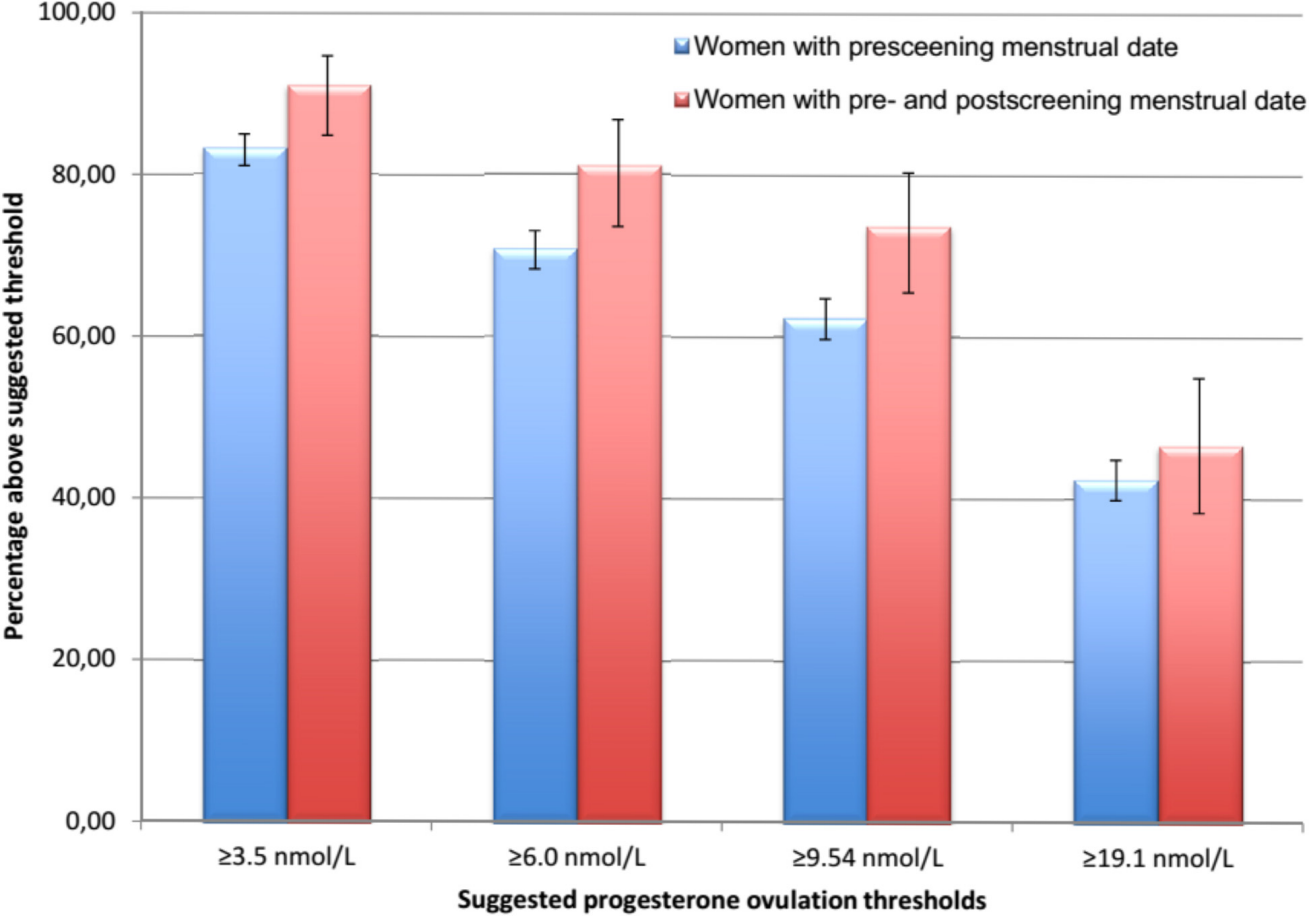
Bar graph of the 1545 women from the HUNT3 (Norway) ovulation study with progesterone levels during cycle days in the presumed luteal phase (cycle days 14 to -3 before usual cycle length) showing the percentage of women that were ovulatory using different threshold serum progesterone levels and by whether they reported the prescreening date menstrual flow started (LMP, cross-hatched bars, n = 1412) or were in a sub-cohort reporting both the LMP and post-screening menstrual flow dates (NMP, open bars, n = 133). Differences between ovulatory percentages in the two cohorts were significant for progesterone thresholds of ≥3.5, ≥8.0 and ≥9.54 nmol/L.

Examination of alternate progesterone thresholds for the diagnosis of ovulation among the 1545 women who were in the presumed luteal phase showed that the percentage classified as ovulatory declined as the potential serum progesterone threshold levels increased (**Fig 3).** Using the serum progesterone threshold of ≥19.1 nmol/L, only 45% were ovulatory; with a threshold of ≥3.5 nmol/L, 84% were ovulatory. Also, the proportion of women with progesterone levels above the various thresholds was higher in the sub-cohort reporting both NMP and LMP (n = 133) versus only LMP (n = 1412) (**Fig 3**) for all threshold levels except ≥19.1 nmol/L.

Predictors of ovulation using the progesterone primary outcome threshold of ≥9.5 nmol/L by univariable and multivariable logistic regressions are shown in **Table 3**. In univariable analysis, the odds ratio for being ovulatory was lowest in the youngest portion of the cohort. Those with progesterone measured on cycle days ≥25 were less likely to be ovulatory, as were nulliparous women and those with both higher and lower serum estradiol values. In the multivariable model adjusted for age, cycle day, estradiol level and parity, and including the cycle day x estradiol interaction term, significant ovulation predictors related to age, parity and cycle days all became non-significant. However, the estradiol level lowest and highest categories remained important predictors of *anovulation* with an inverse U-shaped pattern. Serum estradiol levels ≤120.0 pmol/L and ≥500.0 pmol/L were both associated with very low odds for presumed ovulation. The interaction term between cycle day and its corresponding estradiol level was significant (p<0.05).

**Table 3:** Univariable and multivariable logistic regression of the likelihood of ovulation in spontaneously (without hormonal contraception) and regularly menstruating women aged 20–49.9 based on a serum progesterone level of ≥9.54 nmol/L in HUNT3 (Norway)^#^.

		Serum Progesterone ≥9.54 nmol/L					
				Univariable analysis		Multivariable analysis^	
		N	%	OR	95% CI	OR	95% CI
**Age** ^	<30	50	50.5	**0.51**	**0.31 to 0.84**	0.60	0.32 to 1.11
(years)	30–34	115	66.9	1.0	Ref.	1.0	Ref.
	35–39	215	66.2	0.97	0.66 to 1.43	0.79	0.51 to 1.24
	40–44	314	64.7	0.91	0.63 to 1.32	0.80	0.53 to 1.23
	45–49	284	61.2	0.78	0.54 to 1.13	0.66	0.43 to 1.02
**Cycle day** ^	≤16	219	66.6	1.0	Ref.	1.0	Ref.
(since first	17–20	243	59.7	0.74	0.55 to 1.01	0.75	0.47 to 1.18
day of LMP)	21–24	288	68.6	1.10	0.81 to 1.49	1.31	0.81 to 2.12
	≥25	228	58.6	**0.71**	**0.52 to 0.96**	0.85	0.54 to 1.36
**Estradiol** ^	≤120.0.	21	14.0	**0.07**	**0.05 to 0.12**	**0.29**	**0.11 to 0.81**
(pmol/L)	121–299	486	68.6	1.0	Ref.	1.0	Ref.
	300–499	398	79.0	1.72	12.31 to 2.24	1.58	0.90 to 2.78
	≥500	73	39.9	**0.30**	**0.22 to 0.42**	**0.18**	**0.08 to 0.39**
**Parous** ^	Yes	946	63.8	1.0	Ref.	1.0	Ref.
	No	32	50.8	**0.59**	**0.35 to 0.97**	0.62	0.34 to 1.14
**BMI**	<25	441	64.2	1.0	Ref		
(kg/m^2^)	25–29.9	371	64.6	1.02	0.81 to 1.29		
	30+	165	58.5	0.79	0.59 to 1.05		
**Smoking**	Never	460	64.9	1.0	Ref.		
	Prior	218	60.1	0.81	0.63 to 1.06		
	Current	289	63.2	0.93	0.73 to 1.19		
**Physically**	Yes	585	63.1	1.0	Ref.		
**active >2h/w**	No	382	63.3	1.01	0.82 to 1.25		
**Self-reported health**	Good	788	63.5	1.0	Ref.		
	Fair	164	61.9	0.93	0.71 to 1.22		

^#^The interaction term (cycle day x estradiol, p<0.05) is included in the multivariable model.

Statistically important relationships are shown in **bold**.

**^**Variables significant in univariable models that were included in the multivariable model.

Logistic regression was also performed with lower and higher potential serum progesterone thresholds (≥3.5, ≥8.0 and ≥19.1 nmol/L) as outcomes (data not shown). Briefly, for all progesterone thresholds, serum estradiol was statistically significantly associated with ovulation and showed the same inverse U-shape as reported in **Table 3**. In an adjusted model for evidence of ovulation with a progesterone threshold of ≥8.0 nmol/L as outcome, age also showed an inverse U-shaped association. The interaction term “cycle day x serum estradiol” was also statistically significant in all these models.

## Discussion

This first investigation of ovulation prevalence in a large random population-based sample showed that ovulation point prevalence was 63 to 74 percent using an accepted and validated serum progesterone threshold of ≥9.54 nmol/L in women ages 20–49.9 with spontaneous, regular and normal-length menstrual cycles. In this single cycle, 26–37 percent of cycles showed evidence for anovulation based on a lower than threshold progesterone level despite the expectation that regularly menstruating premenopausal women with normal cycle lengths would always or inevitably be ovulatory [3]. The null hypothesis was rejected.

Those women in the presumed luteal phase who did and did not show evidence of ovulation were virtually identical; this suggests *spontaneous* or sporadic rather than *chronic* anovulation. Based on these and other data [11,20] we now postulate that anovulation is something that intermittently occurs in all or most women [19]. *Prospective* population-based data are now needed to ascertain the within-woman variation in ovulation over time and the incidence of anovulation in women initially documented to be ovulatory in several consecutive cycles.

The prevalence of silent anovulation in over a third of clinically normal menstrual cycles within a large population appears to mandate a new understanding of women’s reproductive physiology. This ovulatory variability perhaps occurs because each menstrual cycle’s hormone levels are created by a single, uniquely stimulated follicle (rather than by the whole ovary or gland as is usual in endocrinology). In addition, each follicle is stimulated to egg-release under tight hypothalamic-pituitary hormonal feedback controls with multiple hypothalamic and limbic inputs. This coordinated ovulation feedback creates a sensitive, adaptive system to allow temporary reproductive suppression during duress [11]. Limbic system affective/emotional and nutritional feedbacks into the hypothalamic-pituitary-ovarian axis occur through the negative influence of increased corticotrophin releasing hormone on gonadotrophin-releasing hormone in response to stressors [11,28,29] often with different threats acting synergistically [28].

Data suggest that ovulation suppression is the most common reproductive adaptation to various stressors [30]. For example, ovulatory disturbances (anovulation and short luteal phases) within regular menstrual cycles in normal-weight women are associated with cognitive dietary restraint [31]; the higher cortisol levels observed in those with higher restraint scores suggests that this attitude toward food and eating, despite lack of weight abnormalities or changes, is intrinsically stressful [32]. Likewise, women with early miscarriages have higher cortisol levels than do women who carry pregnancies to term [33]; first trimester miscarriages are also associated with lower serum progesterone levels, higher self-reported stresses and lower body weights [34].

Progesterone values from different populations differ even when measured using the same methodology and within conception cycles [35]. Also, within one geographic ethic group, women who are advantaged (in health care, education and socioeconomic status) appear to have higher progesterone levels than women who are disadvantaged [36]. Seasons in the Northern hemisphere have also been associated with ovulatory disturbances; these season-related ovulatory disturbances increase when concurrent with increased work/energetic demands [23]. Circumannual changes are related to reproduction through light-dark cycles and pineal melatonin signaling. Thus there are multiple regulatory influences that may decrease the prevalence of ovulation.

It is increasingly evident that silent anovulation within clinically normal menstrual cycles is relevant for women’s health as well as for fertility. Subclinical ovulatory disturbances are associated with annual increased spinal bone losses of -0.86% [19] even when estradiol levels are normal [10]. Increased bone formation through progesterone’s receptor-based osteoblast stimulating actions [37] is needed to prevent bone loss [19]. Ovulatory disturbances are also related to women’s risks for later-life heart disease [20,38,39] and likely also to breast [21] as well as endometrial cancer risks [22].

The anovulation rate in regular menstrual cycles found in this study is similar to results using different ovulation assessment methods including urinary PdG in a nested within population-based two-cycle study in 65 women by Sowers [24] or in 180 women reported by Gorgels [20]. Ovulation point prevalence in such a large population-based sample has not previously been reported.

This study, although in a large cohort, has some limitations including that it is cross-sectional, studied a single cycle, was primarily in white women and, of necessity used an indirect measure of ovulation. Despite the fact that a progesterone ovulatory threshold of 9.54 nmol/L has not been validated against direct visualization of an egg being extruded from the ovary, or serial ultrasounds, it was the spontaneous recommendation of the majority of reproductive experts and two studies have shown this value to predict progesterone-specific secretory endometrial transformation [15,26]. The mean age of the cohort was regrettably in the early 40s; younger women in this rural county must leave to obtain post-secondary training and education and also were more likely to be excluded because they are using hormonal contraception. However, included women were all premenopausal (with regular, normal-length cycles); they are not in the menopausal transition or perimenopause based on recent reproductive aging criteria [40]. The best evidence for an appropriate progesterone threshold (≥9.54 nmol/L) [15,26] and cycle days were used. Although latitude may have influenced these data, they were likely collected randomly across all seasons and thus biases related to light-dark cycles appear unlikely.

This study also has many strengths. We presented results using an array of potential progesterone thresholds, analyzed evidence of ovulation only in those reporting usual cycle lengths of 21–35 days that are considered normal cycle lengths. In addition, we performed a sensitivity analysis in women who knew cycle day-one dates of both menstrual cycles bracketing the date of their serum sample. Other strengths are our population-based cohort, that sampling was random across cycles and that the parent study was related to *general* health. Given that each participant has a unique Norwegian identity number, those who did and did not ovulate in that cycle can be observed prospectively through linkage to national and local disease-specific and mortality registries for the development of diseases such as osteoporosis that are related to ovulation disturbances.

## Conclusions

This first large population-based assessment of indirect evidence for ovulation’s point prevalence using as a primary outcome a serum progesterone threshold of ≥9.54 nmol/L but also assessing a spectrum of threshold values in a main and a sub-cohort shows that anovulation likely occurs in more than a third of all clinically normal menstrual cycles. We also noted that those with/without ovulation in that single cycle differed only minimally. Given increasing evidence that silent ovulatory disturbances within clinically normal cycles are associated with health risks [19], it is important that these data are replicated in population-based samples of women of differing racial and ethnic origins and living at different latitudes.

## Supporting Information

S1 Protocol This supplement describes the protocol related to choosing of the subcohort for whom we had both the start of the last menstrual flow and of the next menstrual flow. (DOCX)

S2 Protocol Serum Documentation Protocol. This supplement describes the handling of the serum after the sample was obtained and the details about measurements of progesterone and estradiol.
(DOCX)
