# Supplementary material for: Ovulation Prevalence in Women with Spontaneous Normal-Length Menstrual Cycles – A Population-Based Cohort from HUNT3, Norway
Source: PLoS One. 2015 Aug 20;10(8):e0134473. doi: 10.1371/journal.pone.0134473 (PMC4546331; doi:10.1371/journal.pone.0134473)
Supplement: S1 Protocol — (DOCX) [file pone.0134473.s001.docx]

**S 1-Protocol**

Eligible women ages 20-49.9 from HUNT3 participating in the Molimina Sub-study were provided a postage-free means of returning the date of the start of their next menstrual flow (NMP). They were chosen if they met any of the following criteria:

- 1. Previous participation in Young-HUNT 1995-97 (from HUNT2—those ages 13-19)
  2. Affirmative answers in HUNT 3 questionnaires indicating having asthma or chronic obstructive pulmonary disease, use of asthma medication(s) in the last five years or attacks of wheezing or breathlessness in the last 12 months
  3. A 10% random sample of all premenopausal women participants
