# Supplementary material for: Ovulation Prevalence in Women with Spontaneous Normal-Length Menstrual Cycles – A Population-Based Cohort from HUNT3, Norway
Source: PLoS One. 2015 Aug 20;10(8):e0134473. doi: 10.1371/journal.pone.0134473 (PMC4546331; doi:10.1371/journal.pone.0134473)
Supplement: S2 Protocol — This supplement describes the handling of the serum after the sample was obtained and the details about measurements of progesterone and estradiol. (DOCX) [file pone.0134473.s002.docx]

**Supplemental Material—Ovulation Prevalence-HUNT3**

**S 2-Serum Documentation Protocol**

Blood-samples were collected into Vacutainer™ 10 ml tubes with clot-gel. After coagulation at room temperature and centrifugation, the vials were kept at low temperature (4 ̊-8 ̊ C) during transportation to the Biobank. Time from collection until the sample was processed into aliquots for freezing was less than 24 hours. Serum was stored in air-tight polypropylene (free from hormonal interference) vials at -80 ̊ C from collection in 2006-2008 until analysis in 2010.

For the hormonal measurements, one serum aliquot was gently thawed, then mixed for 20 minutes before analysis. The quantitative determination of progesterone and estradiol was performed with Liaison® Analyzer from DiaSorin® (Sundbyberg, Sweden). Progesterone and estradiol were measured by a direct competitive chemiluminescence immunoassay; in a photomultiplier, the light signal was subsequently measured as relative light units that are inversely proportional to concentration of each hormone. Progesterone had an analytical range of 1.21-126.3 nmol/L (1 ng/mL = 3.18 nmol/L), with interassay coefficients of variation (CV) of 4.6% at 75 nmol/L and 11.4% at 5.0 nmol/L. Manufacturer-reported normal range for progesterone in the follicular phase was <1.3 – 8.0 pmol/L and for the luteal phase was 3.8 to 79.0 nmol/L. Estradiol had an analytical range of 44 – 4034 pmol/L (1 pg/mL= 3.671 pmol/L); it had a CV of 15.6% at 154 pmol/L and a CV 8.5% at 1487 pmol/L with a follicular phase of 44-411 pmol/L and a luteal phase range of 114 – 500 pmol/L.
